# DMBA induced mouse mammary tumors display high incidence of activating *Pik3ca^H1047^* and loss of function *Pten* mutations

**DOI:** 10.18632/oncotarget.11733

**Published:** 2016-08-31

**Authors:** Martín C. Abba, Yi Zhong, Jaeho Lee, Hyunsuk Kil, Yue Lu, Yoko Takata, Melissa S. Simper, Sally Gaddis, Jianjun Shen, C. Marcelo Aldaz

**Affiliations:** ^1^ CINIBA, Facultad de Ciencias Médicas, Universidad Nacional de La Plata, Argentina; ^2^ Department of Epigenetics and Molecular Carcinogenesis, University of Texas, M.D. Anderson Cancer Center, Smithville, TX, USA; ^3^ CAETI, Facultad de Tecnología Informática, Universidad Abierta Interamericana (UAI), Argentina

**Keywords:** mammary tumors, DMBA, MPA, Pik3ca, Pten

## Abstract

Controversy always existed on the utility of chemically induced mouse mammary carcinogenesis models as valid equivalents for the study of human breast cancer. Here, we performed whole exome and RNA sequencing on long latency mammary tumors (218 ± 27 days) induced by the carcinogen 7,12-Dimethylbenzathracene (DMBA) and short latency tumors (65 ± 11 days) induced by the progestin Medroxyprogesterone Acetate (MPA) plus DMBA in CD2F1 mice. Long latency tumors displayed a high frequency of *Pi3kca* and/or *Pten* mutations detected in 11 of 13 (85%) long latency cases (14/22, 64% overall). Eighty-two percent (9/11) of tumors carried the *Pik3ca* H1047L/R hot-spot mutation, as frequently found in human breast cancer. These tumors were luminal-like and mostly ER/PR+, as in humans. Transcriptome profiling indicated a significant activation of the PI3K-Akt pathway (p=3.82e-6). On the other hand MPA+DMBA induced short latency tumors displayed mutations in cancer drivers not commonly found mutated in human breast cancer (e.g. *Hras* and *Apc*). These tumors were mostly basal-like and MPA exposure led to *Rankl* overexpression (60 fold induction) and immunosuppressive gene expression signatures. In summary, long latency DMBA induced mouse mammary tumors reproduce the molecular profile of human luminal breast carcinomas representing an excellent preclinical model for the testing of PIK3CA/Akt/mTOR pathway inhibitory therapies and a good platform for the developing of additional preclinical tools such as syngeneic transplants in immunocompetent hosts.

## INTRODUCTION

Chemically induced rodent models of mammary cancer have been extensively used over the years to emulate human breast carcinogenesis. All models in mice and rats have specific advantages and limitations. Mammary tumors can be induced in susceptible rat strains after single doses of carcinogens such as DMBA or nitrosomethylurea (NMU). Rat tumors are not extremely invasive beyond the mammary fat pad, have short latency, seldom metastasize and are highly hormone-dependent and for that reason were widely used as models of estrogen dependent breast cancers [1]. On the other hand most mouse strains are far more resistant than rats to chemical induced mammary gland carcinogenesis, typically requiring multiple doses of carcinogens such as DMBA and developing only after a long latency. Three decades ago, it was reported that the progestin medroxyprogesterone acetate (MPA) at very high doses was carcinogenic in the mouse mammary gland [2]. In 1996 we observed that in our hands MPA was not carcinogenic per se but in combination with DMBA mouse mammary tumors developed with a much shorter latency and requiring a lower number of DMBA doses [3].

In terms of the molecular profiles of chemically induced mammary tumors in rodents, not much has been learned beyond the early work of Barbacid and coworkers demonstrating the signature activation of the *Hras* oncogene in NMU induced rat tumors [4, 5]. Hras activation was also reported but with less frequency in DMBA induced tumors both in rats and mice [6–8]. Since HRAS activation is very uncommon in human breast cancer the lingering question always remained on how useful were the chemically induced mammary cancers in rodents as models to study the molecular biology of human breast cancer.

To the best of our knowledge no study to date has analyzed comprehensively the mutational profile of chemically induced mouse mammary tumors in order to understand how closely these models reproduce human breast cancer molecular profiles. To this end we comprehensively analyzed the mutational and transcriptomic profile of DMBA and MPA-DMBA induced mouse mammary tumors by means of exome and RNA sequencing.

## RESULTS AND DISCUSSION

### High mutation rates in short latency MPA+DMBA induced mouse mammary tumors

Exome-Seq data on 22 mouse mammary tumors indicated a median of 75% targeted genome loci having at least 40X coverage. Overall we identified 25898 single base substitutions including 18104 non-synonymous single nucleotide variants (SNVs), 6068 synonymous, 1674 stop-gain and 52 stop-loss SNVs (Supplementary Data 1). We also detected 152 frameshift deletions, 135 frameshift insertions, and 145 non-frameshift deletion/insertion events (Supplementary Data 2).

**Figure 1:**
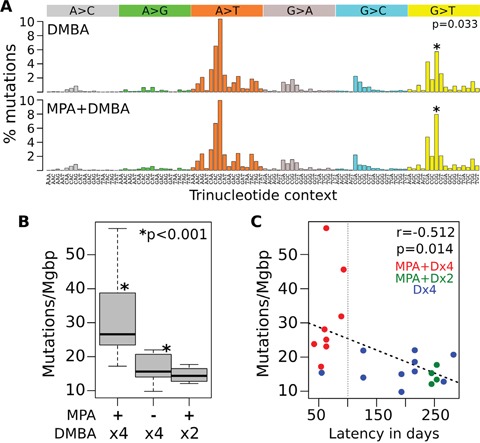
Mutational profiles of chemically induced mouse mammary tumors **A.** Mutational signature of DMBA alone and MPA+DMBA combined treatment. The mutational signature is displayed using a 96 substitution classification defined by the substitution class and the sequence context immediately 5′ and 3′ to the mutated base. The probability bars for each of the six types of substitutions as well as the mutated bases are displayed in different colors. Y-axis represent the percentage of mutations attributed to a specific mutation type corrected by the frequency of its corresponding trinucleotide across the mouse genome. **B.** Comparison of mutation rates per Mgbp among treatments. Note the much higher mutation rate found in MPA+Dx4 generated tumors. **C.** Correlation analysis of mutations per Mgbp and tumor latency in days among tumors. All but one of the short latency tumors were generated by the MPA+Dx4 protocol (red dots).

Almost 64% of the SNVs were A>T:T>A transversions followed by 16% of G>T:C>A substitutions. These data are in agreement with previous studies demonstrating that A>T:T>A and G>T:C>A transversions were the most predominant types of base pair substitutions (44% and 24% respectively) induced by DMBA in rat mammary tissue [9]. Mutational signature analysis allowed us to identify a highly significant 5′-flanking cytidine bias and 3′-flanking cytidine/guanosine bias for A>T transversions in the DMBA-induced tumors (Figure 1A). Addition of MPA treatment to the DMBA protocol appears to slightly modify the overall DMBA mutational signature increasing the frequency of G>T transversions in a 5′-CGG-3′ context compared with DMBA treatment alone (p=0.033, Figure 1A).

The total mutation rate across tumors was 21.7 mutations per Mbp on average with a range of 9.8 to 57.7 mutations per Mbp, indicating that some mouse mammary tumors have significantly higher mutation rates than others. In this sense, MPA+Dx4 (see Methods for nomenclature of treatment groups) derived tumors showed a statistically significant increased rate of mutations/Mbp compared with Dx4 and MPA+Dx2 derived tumors (p<0.001, Figure 1B). More importantly, the highest mutation rate (average of 30 ± 9 mutations/Mbp) was identified in short latency tumors (< 100 days for tumor development with an average of 65 ± 11 days), almost all these tumors derived from the MPA+Dx4 treatment group (red dots in Figure 1C).

### Specific cancer driver genes mutation profiles differentiate short vs. long latency tumors

**Figure 2:**
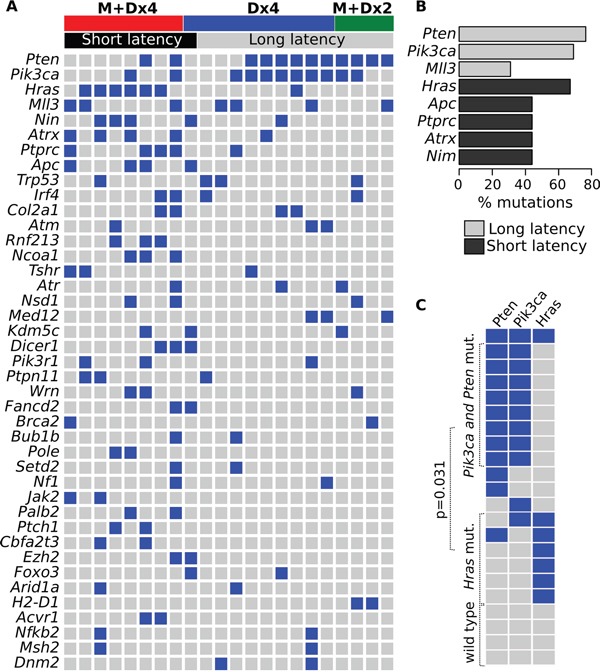
Cancer driver gene mutations in chemically induced mouse mammary tumors **A.** Driver and co-driver mutations in tumor samples. Each column represents one tumor case and each row represents the number of mutations in each gene per tumor. Blue squares, + mutation. **B.** Distribution of the most prevalent driver/co-driver mutations among short (black bars) and long latency (gray bars) chemically induced tumors. **C.** The CoMEt test was employed to identify mutually exclusive events between *Pik3ca*/*Pten* and *Hras* mutations across the 22 tumors analyzed. As can be observed *Pi3ca/Pten* mutations frequently co-occurred in the same tumors and were mostly mutually exclusive with *Hras* mutations.

All tumors displayed mutations affecting multiple targets described as cancer driver genes or potential drivers as defined by the COSMIC Cancer Gene Census [10]. Due to space limitations in Figure 2A we display only cancer driver genes that were found mutated in a minimum of two tumors. Among these, we detected mutations affecting *Pten* in 12 of 22 (55%), *Pik3ca* in 11 of 22 mammary tumors (50%), *Hras* in 7 of 22 (32%), *Mll3* in 7 of 22 (32%) and others such as *Nim*, *Atrx*, and *Ptprc* in 5 of 22 each one (i.e. 23% of cases mutated for each gene) and *Apc, Trp53, Irf4* and *Col2a1* were found mutated in 18% of cases each one (4 of 22 of cases mutated for each gene). Interestingly, we identified a very distinct pattern on the mutational profile of cancer drivers genes clearly differentiating tumors that develop with short latency from those with long latency. The short latency tumors (< 100 days for tumor development) produced mostly by the MPA+Dx4 protocol, were characterized by the predominant presence of mutations affecting the *Hras* oncogene in 6 of 9 (67%) of such early tumors (Figure 2B). Activating *Hras* mutations are well known consequences of exposure to mutagens such as DMBA and other polycyclic aromatic hydrocarbons that have predilection for generating DNA adducts resulting in A>T:T>A transversions as mentioned above and as demonstrated in multiple carcinogenesis models including mammary gland [9]. All of the tumors with *Hras* mutations (7 of 7 in Figure 2) presented the characteristic CAA to CTA mutation affecting codon 61 of *Hras* leading to the Q61L activating mutation.

On the other hand and interestingly, long latency tumors were characterized by a high prevalence of *Pik3ca* and *Pten* mutations (11 of 13 cases, 85%) and only one case displaying an *Hras* mutation (Figure 2B) (see below for further discussion). In addition, we determined that *Pik3ca* and *Pten* mutations behave as mutually exclusive events with respect to *Hras* mutations (CoMEt p-value = 0.031) (Figure 2C).

### Effects of medroxyprogesterone on transcriptomic profiles associated with short latency tumors

**Figure 3:**
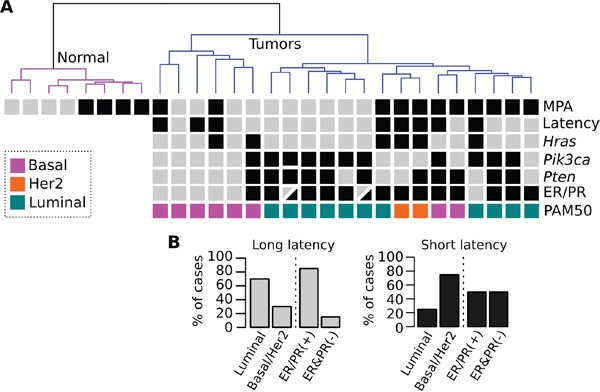
Hierarchical clustering of normal and tumor samples based on RNA-Seq profiles **A.** The first row represent tumors derived from DMBA (gray boxes) or MPA+DMBA combine protocols (black boxes). The second row represents long (gray boxes) and short latency (black boxes) tumors. The third and fourth rows represent tumors that carry *Hras* and *Pik3ca* mutations (black boxes). The fifth row indicates the ER and PR positive (black boxes) and negative (gray boxes) cases according to immunohistochemistry results. Tumor intrinsic subtypes predicted by the PAM50 gene model are highlighted in color codes in the last row. **B.** Intrinsic tumor subtypes according to latency.

**Figure 4:**
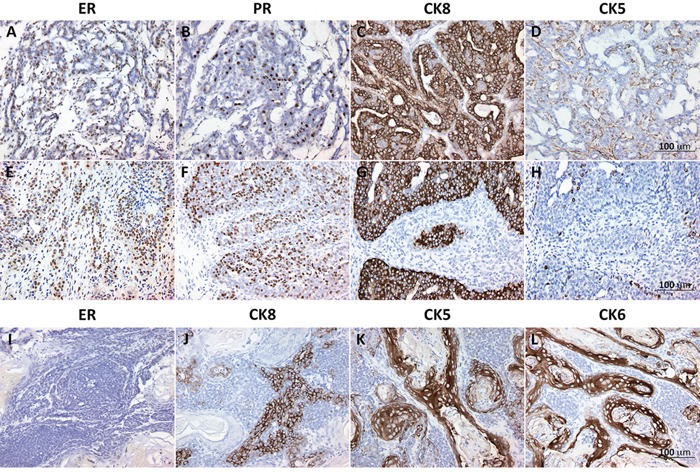
Representative validation of estrogen receptor alpha (ER), progesterone receptor (PR) and luminal and basal cytokeratins (CKs) in mouse mammary tumors Panels **A-D.** representative immunohistochemistry results for long latency (254 ds) tumor T12800. Carcinoma with acinar/glandular differentiation displaying strong ER and PR positivity (brown nuclei in A and B respectively), Strongly positive for luminal CK8 (C) and residual expression in myoepithelial like cells for CK5 (D). This tumor displayed mutations in *Pik3ca* and *Pten* driver genes as per Exome-Seq analyses. Panels **E-F.** short latency (60 ds) tumor T12513. This luminal carcinoma displayed mostly glandular differentiation, strong reactivity for ER, PR and CK8 (**E**-**G.** respectively) and negative for CK5 **H.** Exome-Seq for this case revealed mutations on *Hras* and *Atm* among other potential cancer driver genes. Panels **I-L.** Short latency (55 ds) adenosquamous carcinoma T12521, negative for ER (I) and PR (not shown) expression and with mixed expression of luminal (CK8) and basal cytokeratins CK6/CK5 that strongly stained the squamous differentiated regions.

Unsupervised analysis of RNA-Seq data demonstrates a clear segregation of normal CD2F1 mouse mammary samples and the chemically induced mammary tumors (Figure 3A). In addition, mouse mammary tumors predominantly clustered according to the use or not of MPA (black boxes in first row of Figure 3A). We observed that the intrinsic subtypes determined by immunohistochemistry and predicted by using the PAM50 gene model were significantly associated with each identified cluster. Ninety percent of the DMBA alone (Dx4) induced tumors (grey boxes in first row Figure 3A) were long latency tumors (9 out of 10), 67% of which developed into luminal-like type tumors (Figure 3B). On the other hand, 100% of the MPA+Dx4 induced tumors were short latency cases, 75% of which (6 of 8) classified as basal-like or Her2 subtypes (Figure 3B). Immunohistochemistry analysis of ER/PR, luminal (CK8) and basal (CK5 and CK6) keratins expression of representative short and long latency tumors are shown in Figure 4. Long latency differentiated tumors showed strong ER, PR and CK8 positivity with residual CK5 expression supporting a luminal-like subtype (Figure 4A–4D). While the majority of ER/PR negative short latency tumors showed a mixed expression pattern of basal and luminal cytokeratins with strong reactivity of CK5 and CK6 and low/moderate expression of CK8 (Figure 4I–4L).

**Figure 5:**
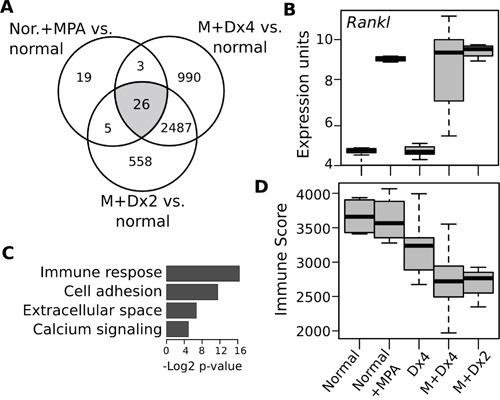
Transcriptomic changes induced by medroxyprogesterone in normal mammary gland and mammary tumors **A.** Venn diagram illustrating the identification of a MPA 26-gene expression signature commonly deregulated in normal and tumors samples. **B.** Functional Gene Ontology enrichment analysis of the MPA-26 gene expression signature. **C.** Box plot of *Rankl* mRNA expression levels comparing normal and chemically induced mouse mammary tumors by the various treatment protocols as indicated at the bottom of D. Note the dramatic increase in *Rankl* expression in normal and tumor samples exposed to MPA treatment. **D.** Comparative immune scores as determined by the ESTIMATE algorithm of normal and tumor samples of various carcinogenesis protocols as indicated. As can be observed carcinogenesis protocols employing MPA treatment (M+Dx4 and M+Dx2) displayed significantly lower immune scores compared with DMBA alone and normal samples (p<0.001).

Comparative transcriptome analysis of MPA treatment in normal mammary epithelium and tumor samples subjected to combined MPA+DMBA protocols allowed us to identify a reduced set of 26 genes modulated by MPA (Figure 5A, Supplementary Data 3). Not surprisingly, the *Rankl* transcript (also known as *Tnfsf11*) was the most up-regulated gene in MPA treated normal (Log2FC = 5.4; p<0.0001) and tumor (Log2FC = 6.1; p<0.0001) samples (Figure 5B). In previous studies it was demonstrated that MPA induces a 3000-fold increase of *RANKL* mRNA expression in mammary epithelial cells leading to the activation of the RANK/RANKL signaling cascade driving proliferation of mammary epithelial cells [11, 12]. Moreover, previous studies also demonstrated that progesterone triggers mammary proliferation via two distinct mechanisms: an early RANKL-independent direct mitogenic effect on PR-positive cells followed by a wave of greater proliferation mediated by the paracrine effect of RANKL on PR-negative cells [13]. It is also known that the RANK/RANKL signaling pathway plays a major role not only in the development of normal mammary gland during pregnacy, but also has been identified as an important modulator of mammary stem-cell expansion as well as being a critical player in breast cancer metastasis development [14, 15].

Interestingly, functional enrichment analysis identified “immune response”, “cell adhesion” and “degradation of ECM” to be highly associated with the 26 MPA-modulated genes (Figure 5C). Supporting the functional enrichment analysis, a gene expression signature–based method known as ESTIMATE predicted decreased immune scores on MPA-DMBA induced tumors compare with DMBA alone (p<0.01) and normal tissues (p<0.001) (Figure 5D). These findings are in agreement by abundant literature reporting a critical role of progesterone in modulating immune tolerance to fetal development during pregnancy [16]. Progesterone actively affects various types of immune cells such as T and B cells, NK cells, macrophages and dendritic cells [17], mostly inhibiting activation, differentiation and motility of the various immune cell types. Furthermore, it has been shown that progesterone induces the differentiation of T cells into immune-suppressive T regulatory cells (Tregs) [18] and in turn activated Tregs are known to be critical in the development of a tumor-associated immunosuppressive phenotype [19]. Importantly it has been recently demonstrated that MPA (Depo-Provera™) suppresses innate and adaptive immune mechanisms, including inhibiting the production of INFG and IL-2, IL-4, IL-6, IL-12, TNFα, MIP-1α and various other cytokines in circulating blood mononuclear cells and activated T cells, and these dramatic immunosuppressive effects were associated with the increase of HIV infections in patients receiving MPA as contraceptive [20].

Taken together, the present study suggests that MPA treatment accelerates mammary tumorigenesis in this and other rodent mammary tumor models via *Rankl*-mediated proliferative expansion of the DMBA ‘initiated’, *e.g. Hras* or *Apc* mutated mammary epithelial cells, via direct and paracrine mechanisms while additionally favoring the development of an immunosuppressive (anergic) microenvironment that facilitates the expansion of the highly proliferative cells ultimately leading to the rapid development of mammary tumors (*i.e.* short-latency tumors).

### High incidence of *Pik3ca* and *Pten* mutations are characteristics of long latency mammary tumors

As mentioned above, we detected *Pik3ca* and/or *Pten* mutations in 64% (14 of 22) of the mammary tumors analyzed. However, we observed that long latency tumors (>100 days for tumor development, Figure 1C), mostly derived from the Dx4 treatment group, were characterized by bearing most of the *Pik3ca* and/or *Pten* mutations (11 of 13 long latency tumors, 85%), contrasting with the short latency tumors characterized by carrying *Hras* driver mutations (67%) (Figure 2A, 2B).

Originally, *PIK3CA* and *PTEN* mutations were thought to be mutually exclusive in various cancers with the exception being endometrial tumors where coexistent mutations of both genes were reported [21, 22]. However in later reports analysis of *PIK3CA* mutation and PTEN protein loss in breast carcinomas showed that both alterations were indeed not mutually exclusive indicating that *PIK3CA* mutations coexist with PTEN loss in ER + breast cancers [23]. In agreement with those observations in our study we detected that indeed *Pik3ca* and *Pten* behave as co-occurring mutations in the chemically induced mouse mammary tumors analyzed (p=0.003, Figure 2C).

Fifty percent of the tumors with *Pten* alterations involved non-synonymous substitution (5 of 12) and frameshift insertion (1 of 12) resulting in stop-gain mutations, the remaining cases (6 of 12) consist in deleterious *Pten* mutations leading to loss-of-function as was predicted by the SIFT algorithm.

**Figure 6:**
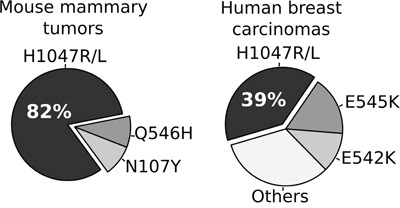
Comparative analysis of the frequency of *Pik3Ca/PIK3CA* hot-spot mutations among chemically induced mouse mammary tumors and human breast carcinomas *PIK3CA* mutational profile in primary breast carcinomas was obtained from the TCGA-BRCA project [24].

The comparative frequency of mutations on the *Pik3ca* gene in our chemically induced mouse mammary tumors and in human invasive breast carcinomas (data obtained from TCGA project, http://www.cbioportal.org/) [24] is shown in Figure 6. These results are revealing, indicating that 82% (9 out of 11) of DMBA-induced tumors carried exactly the same initiating ‘hot-spot’ mutation in *Pik3ca* (H1047L/R), which is by far the most frequent *PIK3CA* mutation reported in human invasive breast carcinomas (138 out of 349 identified mutations, 39.5%). Furthermore, the H1047R mutation was demonstrated to activate PI3K signaling in human mammary epithelial cells and induces tumor formation in nude mice [25].

We consider this a very significant finding considering that *PIK3CA* somatic mutations are detected in over 34% of human breast cancers and there is a need for pre-clinical mouse mammary cancer models that more closely reproduce the histology and biology of the human counterparts for the *in vivo* testing of anti-PIK3CA therapies [reviewed in 26].

### Transcriptomic analysis of long latency mammary tumors revealed an activated PI3K-Akt signaling pathway

**Figure 7:**
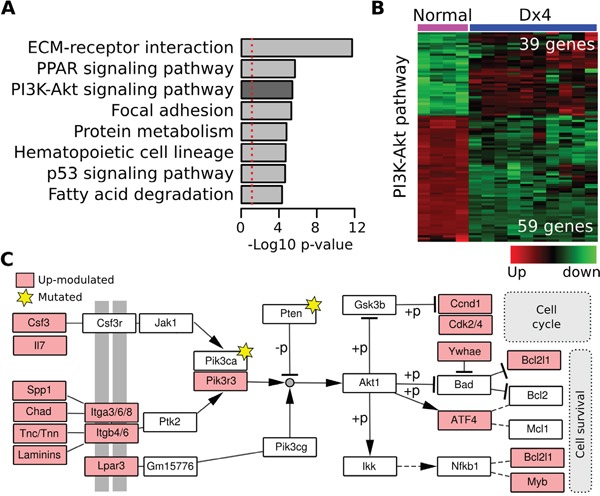
Analysis of differentially expressed transcript in DMBA-induced mouse mammary tumors **A.** Functional enrichment analysis of transcripts differentially expressed among DMBA-induced tumors and normal samples (p-adj < 0.001; Log2FC > 1). **B.** Heat map of 98 PI3K/AKT related genes differentially expressed between DMBA-induced tumors and normal samples. **C.** PI3K/AKT pathway diagram highlighting some of the genes mutated (stars) and up-modulated (colored boxes) in DMBA-induced mouse mammary tumors.

RNA-Seq analysis revealed a group of 3675 differentially expressed genes (FDR < 0.001; logFC > 1, Supplementary Data 3) between normal samples and DMBA (Dx4) induced mammary tumors. Interestingly, genes associated with the Dx4 long latency mammary tumors were strongly related to the modulation of the PI3K-Akt pathway (98 related genes; p=3.82e-6), Focal adhesion (64 genes, p=4.98e-6), ECM-receptor interaction (45 genes, p=1.70e-12), PPAR signaling pathway (33 genes; p=2.03e-6), p53 signaling pathway (27 genes, p=2.26e-5) and metabolic processes (Figure 7A). Among the 98 PI3K-Akt related genes, 39 were up-modulated transcripts and 59 were down-modulated transcripts in Dx4 induced tumors (Figure 7B). More importantly, several of these genes (23 out of 39) were specifically associated with cell cycle and cell survival PI3K-Akt mediated activity through up-modulation of cytokine-receptor interactions, signal transducers (*Pik3r3* over-expression, *Pik3ca* and *Pten* mutations) and multiple downstream effectors such as *Ccnd1*, *Cdk4*, *ATF4* and *Myb* among others.

In this sense, *Spp1* (also know as *Osteopontin, Opn*) was the top up-regulated gene in Dx4 induced tumors (log2FC = 6.85; p-adj < 0.0001). OPN is a multi-functional cytokine that binds to cell surface receptors, such as CD44 and integrins, which are involved in immune response, cell survival, adhesion and migration. OPN has been previously associated with tumor formation, progression and breast cancer metastasis [27]. Recently has been demonstrated that OPN abrogation in breast cancer cells leads to the inhibition of cell migration and invasion through inhibition of PI3K-Akt-mTOR signaling pathway [28]. It is important to note that several integrins related with PI3K-Akt signaling (*e.g.: Itgb4/6* and *Itga3/6/8*) were also up-modulated in Dx4 chemically induced tumors. Together, our results suggest that the overexpresion of *OPN*, several PI3K-Akt related binding receptor and several down-stream modulators including *Pik3r3*, *Ccnd1* and *Cdk4* among others could be part of a positive autoregulatory loop controlling tumor growth in long latency chemically induced tumors.

### Concluding remarks

The comprehensive molecular characterization of chemically induced mouse mammary tumors allowed us to define mutational and transcriptomic signatures driving the DMBA and MPA induced carcinogenic processes. Short latency tumors derived mostly by the MPA+Dx4 protocol, were characterized by high mutational burden affecting multiple cancer driver genes including the predominant frequency of mutations affecting the *Hras* oncogene. Additionally, transcriptomic analysis suggests that MPA exposure accelerates mammary tumorigenesis not only via *Rankl*-mediated proliferative expansion of the carcinogen ‘initiated’ mammary epithelial cells carrying cancer driver mutations such as *Hras* activating and *Apc* loss of function mutations, but also by generating an immunosuppressive tumor microenvironment that likely facilitates the development of short-latency basal-like mammary tumors. On the other hand, DMBA-induced long-latency tumors were characterized by A>T and G>T substitutions frequently affecting the *Pik3ca* and *Pten* cancer driver genes. More importantly, 82% of the cases with *Pik3ca* missense mutations displayed the characteristic kinase domain H1047L/R ‘hot-spot’ activating mutation that has been reported as the most frequent *PIK3CA* mutation in human breast cancers. Furthermore, transcriptomic analysis of the DMBA induced tumors pointed to PI3K-Akt signaling pathway activation as extremely common in long latency luminal-like tumors.

As recently reviewed [26], there is a need for Pik3ca mouse mammary tumor models that more closely reproduce the histology and biology of the human breast cancer *PIK3CA* mutated counterparts. Thus, the DMBA induced mouse mammary tumor model appears as an excellent system to provide a better understanding of *Pik3ca/Pten* mutant driven mammary cancer pathogenesis but also as a prime candidate for further developing pre-clinical models (e.g. syngeneic transplant lines) for the *in vivo* testing of Pik3ca/Akt/mTor pathway inhibitory therapies.

## MATERIALS AND METHODS

### Mouse mammary samples

Thirty fresh-frozen CD2F1 (BALB/c x DBA/2) mouse mammary normal and tumor samples were obtained from a tumor bank at the Aldaz lab from studies performed and reported 20 years ago in 1996 [3]. Briefly, a total of 22 mammary tumors were analyzed by whole exome-seq and RNA-seq. Twelve of these tumors were derived from MPA-DMBA treatment groups, meaning that female mice received two implanted subcutaneous pellets of compressed MPA (20 mg each pellet, standard release) at 6 wks of age followed by either 2 or 4 doses of DMBA administered intragastrically (1 mg/dose) starting at week 9 and referred as MPA+Dx2 and MPA+Dx4 respectively. In addition 10 mammary tumors were obtained from female mice that received only 4 doses of DMBA (1 mg/dose) as treatment (i.e. Dx4 group). All the aforementioned treatment groups, detailed protocols and comparative outcomes were previously described [3]. We also analyzed a group of 8 normal mammary gland samples, 4 derived from 13 wk old female mice subjected to only MPA treatment for 4 weeks (i.e. Normal + MPA group) and 4 derived from 13 week old untreated female mice (i.e. Normal group). The tumor incidence and latency results were retrieved from the previously reported study [3].

Routine histopathologic evaluation was performed on all tumors by means of formalin fixation, paraffin embedding and hematoxylin and eosin stained sections. For tumor classification we followed the recommendations of the Annapolis consensus report for the analysis of mouse mammary tumors [29] (Supplementary Data 4). All samples were analyzed for ER and PR expression by immunohistochemistry (IHC) using standard procedures and tumors were considered positive for expression of either receptor when at least a minimum of 10% of the tumors cells demonstrated clearly detectable nuclear staining (Supplementary Data 4). The staining patterns of CK5 (PRB-160P antibody, BioLegend), CK6 (PRB-169P antibody, BioLegend) and CK8 (TROMA-I antibody, DSHB) keratins were analyzed by IHC to confirm the basal or luminal subtype.

### Exome-seq data analysis

DNA from 22 mouse mammary tumor samples and two normal mouse mammary samples from strain CD2F1 were purified using the DNeasy Blood and Tissue Kit (Qiagen). Only DNA samples with 260/280 ratios greater than 2.0 were processed for library construction using the SPRIworks Fragment Library Kit I (Beckman Coulter). Four libraries were pooled together and processed for exome capture using the NimbleGen SeqCap EZ Developer Library (110624_MM9_exome_L2R_D02_EZ_HX1, Roche), covering ~ 54.3 Mbp of target sequence. 76 nt paired-end sequencing was performed using an Illumina HiSeq2000 platform at our Department's NGS Facility. Image analysis, base-calling, and error calibration were performed using Illumina's Genome analysis pipeline. Sequencing was performed reaching an average depth of 40X per sample. Sequenced 76 bp tags were aligned against the mouse reference genome (GRCm38/mm10) using BWA v0.7.3 and marked for duplicates using Picard v1.88 (http://picard.sourceforge.net/). Tumors were called against normal DNA isolated from two wild-type CD2F1 mice. Subsequently, single-nucleotide variants were identified using MuTect v1.1.4 [30]. Identified variants were annotated using ANNOVAR [31] and Ensembl VEP (http://www.ensembl.org/Tools/VEP) to compute the SIFT scores and VarScan software (http://dkoboldt.github.io/varscan/) was employed for indels detection. Detected somatic mutations were further filtered by focusing only on those genes considered cancer driver genes as defined by the COSMIC Cancer Gene Census database (http://cancer.sanger.ac.uk/census/). The Comet Exact Test (CoMEt) algorithm was employed to identify mutually exclusive mutations [32].

Mutational spectra analysis of DMBA and MPA+DMBA treatments was determined as previously described for human tumors [33]. Briefly, SNVs detected among 22 tumors were annotated by the 96 possible trinucleotide context substitutions (6 types of substitutions × 4 possible flanking 5′ bases × 4 possible flanking 3′ bases) and summed in each tumor according DMBA or MPA+DMBA groups. These counts were converted to per group proportions and subsequently adjusted for the trinucleotide frequency in the mouse genome.

### RNA-seq data analysis

RNA was isolated and purified using TRIzol reagent (Life Technologies) and RNeasy mini kit (Qiagen). RNA concentration and integrity were measured on an Agilent 2100 Bioanalyzer (Agilent Technologies). Only RNA samples with RNA integrity values (RIN) over 8.0 were considered for subsequent analysis. mRNA from normal mouse mammary and tumor samples were processed for directional mRNA-seq library construction using the ScriptSeq v2 RNA-Seq Library Preparation Kit (Epicentre) according to the manufacturer's protocol. We performed 76 nt paired-end sequencing using an Illumina HiSeq2000 platform and obtained ~23-37 million tags per sample. The short sequenced reads were mapped to the mouse reference genome (mm10) by the splice junction aligner TopHat V2.0.10 [34]. We employed several R/Bioconductor packages to accurately calculate the gene expression abundance at the whole-genome level using the aligned records (BAM files) and to identify differentially expressed genes between normal CD2F1 mouse mammary samples and the chemically induced mammary tumors [35]. Briefly, the number of reads mapped to each gene based on the TxDb.Mmusculus.UCSC.mm10.KnownGene database were counted, reported and annotated using the Rsamtools, GenomicFeatures, GenomicAlignments, and org.Mm.eg.db libraries. Raw datasets have been submitted to NCBI GEO database. To identify differentially expressed genes between normal mammary epithelium and tumor samples, we utilized the DESeq2 test based on the normalized number of counts mapped to each gene [36]. Functional enrichment analyses were performed using the ClueGo Cytoscape's plug-in (http://www.cytoscape.org/) and the InnateDB resource (http://www.innatedb.com/) based on the list of deregulated transcripts between normal and mouse mammary tumors (FDR<0.001; log FC>±1). Data integration and visualization of differentially expressed transcripts were done with R/Bioconductor [35] and the MultiExperiment Viewer software (MeV v4.9) [37]. Intrinsic subtype classification of tumors into Luminal-like, Basal-like, Her2-enriched and Normal-like groups was performed using the 50-gene (PAM50) predictor bioclassifier R script [38]. In addition, we used the ESTIMATE algorithm (Estimation of STromal and Immune cells in Malignant Tumors using Expression data) to infer the immune and stromal components from each tumor sample [39].

## SUPPLEMENTARY DATA, FIGURES AND TABLES










