# Supplementary material for: DMBA induced mouse mammary tumors display high incidence of activating Pik3caH1047 and loss of function Pten mutations
Source: Oncotarget. 2016 Aug 31;7(39):64289–99. doi: 10.18632/oncotarget.11733 (PMC5325442; doi:10.18632/oncotarget.11733)
Supplement: Supplementary file 1 [file oncotarget-07-64289-s001.pdf]

DMBA induced mouse mammary tumors display high incidence of activating *Pik3ca*^{H1047} and loss of function *Pten* mutations

SUPPLEMENTARY DATA

Supplementary Data 1: List of single nucleotide substitutions in mouse mammary tumor samples

See Supplementary File: 1

Supplementary Data 2: List of Indels detected in mouse mammary tumor samples

See Supplementary File: 2

Supplementary Data 3: Differentially expressed transcripts between chemically induced mouse mammary tumors and normal mammary samples.

See Supplementary File: 3

Supplementary Data 4: Description of samples employed for Exome-Seq and RNA-Seq analysis.

See Supplementary File: 4
